# Psychosocial work characteristics and low back pain in daycare (nursery) workers in Japan: a prospective cohort study

**DOI:** 10.1186/s12891-022-06009-x

**Published:** 2022-12-03

**Authors:** Xuliang Shi, Megumi Aoshima, Tadayuki Iida, Shuichi Hiruta, Yuichiro Ono, Atsuhiko Ota

**Affiliations:** 1 Department of Public Health, Fujita University School of Medicine, 1-98 Dengakugakubo, Kutsukake-cho, Toyoake, Aichi 470-1192 Japan; 2 Department of Physical Therapy, Faculty of Health and Welfare, Prefectural University of Hiroshima, 1-1 Gakuen-cho, Mihara, Hiroshima, 723-0053 Japan; 3 Research Center of Health, Physical Fitness, and Sports, Nagoya University, Furo-cho, Chikusa- ku, Nagoya, 464-8601 Japan

**Keywords:** Daycare (nursery school), Effort-reward imbalance, Job strain, Low back pain, Overcommitment to work, Psychosocial work characteristics, Social support

## Abstract

**Background:**

Low back pain (LBP) is one of the most common musculoskeletal problems affecting daycare (nursery) workers. We aimed to identify the psychosocial factors influencing LBP in daycare workers.

**Methods:**

We conducted a prospective cohort study with a one-year observation period. The baseline sample was a convenience sample of 444 daycare workers from 34 daycare facilities in Nagoya, Japan, and its suburbs. All the data were collected through a questionnaire survey. The question “Where are you currently feeling LBP?” was used to determine whether the subjects suffered from LBP. We examined the prospective relationships of the psychosocial work characteristics, i.e., high job strain, low social support, effort-reward imbalance, and overcommitment, at baseline and LBP after one year. We used multiple logistic regression analyses to calculate the odds ratios of psychosocial work characteristics for the persistence and onset of LBP, adjusted for age, sex, body mass index, smoking, employment status, occupation, and working schedule.

**Results:**

At baseline, 270 (60.8%) subjects suffered from LBP. Of 208 who also gave information on LBP one year later, 176 (84.6%) suffered from the persistence of LBP. Low social support at baseline was significantly related to persistent LBP one year later. The incidence of persistent LBP was 89.9% and 80.0% among those with and without low social support at baseline, respectively. The adjusted odds ratio (95% confidence interval) of low social support at baseline for the persistence of LBP was 2.43 (1.01–5.87). Of 150 who were without LBP at baseline and provided information on LBP one year later, 45 (30.0%) suffered from the onset of LBP. None of the psychosocial work characteristics showed significant relationships with the onset of LBP one year later.

**Conclusion:**

Low social support was related to the persistence, but not to the onset of LBP in a prospective cohort analysis among daycare workers in Japan. High job strain, ERI, or overcommitment did not show a significant prospective effect on LBP.

## Introduction

Low back pain (LBP) impacts workers’ health and occupational safety [1, 2]. LBP also impacts workers in daycare facilities (or nursery schools) for preschool children. Previous cross-sectional studies have shown that musculoskeletal disorders are an important factor affecting the careers of daycare teachers [3–6]. In Japan, Tsuboi et al. reported the prevalence of LBP in daycare teachers as much as about 43.0% [7], whereas Yamamoto-Kataoka et al. reported a considerably higher prevalence (83.7%) [8].

Theory-based conceptual models have recently been used to explain the relationship between psychosocial work characteristics and health. The Job Demand Control Support (JDCS) [9, 10] and Effort-Reward Imbalance (ERI) [11, 12] models are well-known models which assess psychosocial work characteristics in different dimensions. These models have been used to test the associations between psychosocial work characteristics and various health concerns. The JDCS model provides three dimensions, i.e., job demand, job control, and social support, to explain the psychosocial work stressors. Job strain was defined as the ratio of psychological demand to decision latitude. High job strain is a condition in which quantitatively high and conflicting demands are combined with little decision authority and skill utilization. Social support is defined as helpful social interactions with supervisors and co-workers in the workplace, which is expected to counteract the effects of a harmful psychosocial work environment. In the ERI model, those who were rewarded insufficiently in terms of money, esteem, opportunities, and job security for their efforts, i.e., demands and obligations, were defined as being in a state of ERI. Overcommitment is a personal trait related to dysfunctional coping methods in response to work-related stressful situations and feelings.

Both physical and psychosocial factors may play an important role in the persistence and onset of musculoskeletal disorders. Previous findings showed that psychosocial work characteristics could be related to the persistence and onset of LBP, although few studies have been conducted for daycare workers. Results of prospective cohort studies [13–17], which included other kinds of workers as the subjects, were not consistent regarding the effect of low social support on the persistence and onset of LBP. Previous studies revealed that high job strain did not affect the persistence of LBP [15, 17] or sick leave due to LBP [14, 16] in other kinds of workers. Koch et al. found that ERI was related to LBP in childcare workers in Germany [18]. Rugulies et al. found the effect of ERI on the onset of LBP in transit vehicle operators [19], while Lapointe et al. failed to find such an effect in video display unit users [20]. No previous study addressed the effect of overcommitment on the persistence or onset of LBP. Eventually, it remained unclear what kind of psychosocial work characteristics contributed to the prognosis of LBP in workers, including daycare workers. Therefore, we aimed to determine the psychosocial work characteristics, as assessed with the JDCS and ERI models, affecting the persistence and onset of LBP among daycare workers by longitudinal analyses.

## Methods

### Design

We employed a prospective cohort design. Baseline data were collected in June 2015. The follow-up survey was carried out one year later, in June 2016.

### Subjects

A convenience sample was used. To recruit the baseline participants, we invited the employees of 36 daycare facilities located in Nagoya City, Japan, and its suburban area. There are 527 authorized daycare facilities in Nagoya City as of April 2022 [21]. Although we did not sum up the exact number of workers in the 36 daycare facilities, we were reported that approximately 20 full-time workers and 20 part-time workers were averagely working in each daycare facility. Therefore, the total number of eligible participants was estimated 1440. To reduce costs, we did not distribute the questionnaire to all workers but only to those who were willing to participate in this study. Of the 36 daycare facilities, 34 had at least one respondent, while 2 had no respondent. The total number of respondents was 446, which resulted in an estimated response rate of approximately 30%. After excluding two participants without a valid response regarding LBP, we analyzed 444 subjects. Of them, 361 people (81.3%) participated in the follow-up survey.

### Study variables

The variables in this study were as follows. All the data were collected through a self-administered questionnaire.

### LBP

#### Definition of LBP

The question “Where are you currently feeling LBP?” was used to determine whether the participants suffered from LBP both in the baseline and follow-up surveys. We provided the following five answer options to specify the location of pain: (1) feeling pain in the lower back only, (2) feeling pain in the lower and upper back, (3) feeling pain that extends to the hips and/or buttocks, (4) feeling pain and numbness that extends to the legs and feet, and (5) feeling pain in the shoulders, neck, and/or arms as well as the lower back. The participants were asked to answer “yes” or “no” to each option. Those who answered “yes” to any option were defined as subjects suffering from LBP. Those who answered “no” to all five options were defined as not suffering from LBP. The subjects who had LBP both in 2015 and 2016 were defined as suffering from persistent LBP. Those who did not have LBP in 2015 but suffered from LBP in 2016 were defined as experiencing the onset of LBP.

#### Pain intensity

Pain intensity was assessed by the numerical rating scale (NRS) score [22, 23]. The NRS is a measurement instrument used to determine the degree of pain that ranges over a continuum of values and cannot be directly measured. In this study, the degree of LBP ranged from 0 to 10, as assessed by the NRS, where 0 indicated that no pain was felt, whereas 10 indicated the worst pain imaginable by the subject.

#### Disability in daily life due to LBP

The Roland-Morris Disability Questionnaire (RDQ) is a scale that was used to examine whether LBP affected daily activities such as standing, walking, sitting, dressing, and working. It comprised 24 items. The responses were “yes” and “no” for each item. The cumulative number of “yes” responses indicated the score, which ranged from 0 to 24. The higher the score, the worse the degree of disability in daily life. In this study, we used the Japanese version of the RDQ [24].

#### Impact of LBP on work

We examined the impact of LBP on work. The subject chose one of the following six options: (1) I cannot work without taking occasional days off; (2) I cannot work without taking an occasional break; (3) It hurts a lot, but I do not need to take a break; (4) I feel slight pain occasionally; (5) I would like a break or day off, but I cannot; and, (6) I do not have severe pain.

### Psychosocial work characteristics

We employed the JDCS and ERI models to assess the psychosocial work characteristics. Both models are theory-based conceptual models for the assessment of adverse psychosocial job stressors.

Using the Japanese version of the Swedish Demand-Control-Support Questionnaire [25], we quantified job strain and social support of the subjects. Job strain was defined as the ratio of psychological demand to decision latitude. Referring to previous studies [26, 27], we defined the subjects in the top quartile of job strain scores as being exposed to high job strain and those with a social support score lower than the median as receiving low social support.

Using the Japanese version of the ERI questionnaire [28], we assessed whether the subjects were experiencing ERI and overcommitment. Those who had an effort/reward ratio score of 1.0 or higher were defined as experiencing ERI [29]. Those in the upper tertile of overcommitment scores were defined as being overcommitted to work.

### General characteristics

We collected information about the subjects’ sex, age, body mass index (BMI), smoking, and working conditions. BMI was calculated using the self-reported heights and weights. The working conditions included employment status (regular employment or not), occupation (teachers, cooks/nutritionists, and others), and working schedule (irregular or not).

### Statistical analysis

First, we assessed the baseline characteristics of the subjects. Then, we examined the prospective relationships between the psychosocial work characteristics at baseline and LBP one year later using the follow-up survey data. We classified the subjects by the presence/absence of LBP at baseline to differentiate the effects of the psychosocial work characteristics on the persistence of LBP from those on the onset of LBP. For the comparisons, after calculating the prevalence of the persistence/onset of LBP, we used multiple logistic regression analyses to calculate the relevant odds ratios, adjusting for age, sex, BMI, smoking, employment status, occupation, and working schedule. As we could not follow-up all the subjects, we compared the baseline characteristics between those who did and did not participate in the 2016 survey. IBM SPSS 28 was used for the statistical calculations. P-values less than 0.05 were regarded as statistically significant.

## Results

### Baseline characteristics

The baseline characteristics of the 444 subjects are shown in Table 1. The mean age was 34.5 years. Approximately 90% were women, teachers, and regularly employed, whereas approximately 80% had an irregular work schedule. LBP was reported by 270 (60.8%) subjects.

Table 2 presents the NRS and RDQ scores and impact on work at baseline, stratified by LBP statuses. Of those with LBP in 2015, the means (standard deviations) of the NRS and RDQ scores were 3.6 (2.0) and 1.7 (2.7), respectively. Approximately three-quarters of them felt slight pain occasionally. Those with the onset of LBP had higher mean NRS scores (1.2 vs. 0.7) and, regarding the impact on work, more frequently complained that “I felt slight pain occasionally” (48.9% vs. 31.0%) than those without LBP in 2015.

**Table 1:** Characteristics of the participants in 2015 (*n*=444)

Variable	n(%)/Mean(SD)
**Sex (Female)**	**398 (89.6%)**
**Age** ^**a**^	**34.5 (11.9)**
**Body mass index (BMI)** ^**b**^	**21.3 (3.0)**
**Current smoker** ^**c**^	**29 (6.5%)**
**Employment status: regular** ^d^	**388 (87.4%)**
**Occupation** ^e^	
** Teacher**	**380 (85.6%)**
** Cook/Nutritionist**	**46 (10.4%)**
** Others**	**17 (3.8%)**
**Working schedule: irregular** ^f^	**363 (81.8%)**
**Work stress**	
** Job strain** ^g^	**0.78 (0.17)**
** Social support** ^h^	**19.8 (3.0)**
** Effort-reward imbalance** ^i^	**36 (8.1%)**
** Overcommitment** ^j^	**14.9(3.3)**
**Low back pain: present**	**270 (60.8%)**

Figures are presented as proportions (%) or means (standard deviations)

The numbers of missing responses: a) 5, b) 15, c) 28, d) 2, e) 1, f) 3, g) 7, h) 8, i) 26, and j) 12

**Table 2:** The numerical rating scale (NRS) score, Roland-Morris Disability Questionnaire (RDQ) score, and impact on work at baseline 2015, stratified by low back pain (LBP)

LBP	Subjects at baseline		LBP status one year later	
	Present (*n*= 270)	Absent (*n*= 174)	Persistent (*n*= 176)	Onset (*n*= 45)
**NRS score**	**3.6 (2.0)** ^a^	**0.7 (1.1)** ^b^	**3.7 (2.1)** ^c^	**1.2 (1.2)**
**RDQ score**	**1.7 (2.7)** ^d^	**0.1 (0.4)** ^e^	**1.8 (2.8)** ^f^	**0.1 (0.5)**
**Impact on work** ^g^				
** I cannot work without taking occasional days off.**	**0**	**0**	**0**	**0**
** I cannot work without taking a break sometimes.**	**6 (2.2%)**	**0**	**5 (2.8%)**	**0**
** It hurts a lot, but I do not need to take a break.**	**45 (16.7%)**	**0**	**32 (18.2%)**	**0**
** I feel slight pain occasionally.**	**201 (74.4%)**	**54 (31.0%)**	**129 (73.3%)**	**22 (48.9%)**
** I would like a break or day off, but I cannot.**	**5 (1.9%)**	**1 (0.6%)**	**4 (2.3%)**	**0**
** I do not have severe pain.**	**11 (4.1%)**	**119 (68.4%)**	**5 (2.8%)**	**23 (51.1%)**

The numbers of missing responses: a) 6, b) 4, c) 4, d) 2, e) 3, and f) 1

^g)^ The number of missing responses: 2 for participants with LBP in 2015 and 1 for those with persistent LBP in 2016

### Longitudinal effect of the psychosocial job characteristics at baseline on LBP after one year

Of the subjects who had LBP at the baseline (*n* = 270), 209 (77.4%) participated in the follow-up survey one year later. Of 208 who gave information on LBP, 176 (84.6%) suffered the persistence of LBP. Only low social support was related to the persistence of LBP (Table 3). Those with low social support at baseline presented a higher prevalence of persistent LBP than those without (89.9% vs. 80.0%). The crude odds ratio (95% confidence interval: CI) was 2.23 (1.00–4.96) (*p* < 0.05). The adjusted odds ratio was not significant when age, sex, BMI, and smoking were adjusted (adjusted odds ratio (AOR) = 2.13, 95% CI: 0.90–5.07, *p* < 0.1), whereas it was significant when the working conditions, i.e., employment status, occupation, and working schedule, were additionally adjusted (AOR = 2.43, 95% CI: 1.01–5.87, *p* < 0.05). The other psychosocial job characteristics did not show significant relationships with persistent LBP one year later.

Of the subjects without LBP at baseline (*n* = 174), 152 (87.4%) participated in the follow-up survey one year later. Of 150 who provided information on LBP one year later, 45 (30.0%) suffered from the onset of LBP. Low social support did not significantly affect the onset of LBP (Table 4). Those with low social support at baseline presented a higher incidence of LBP than those without (37.9% vs. 24.4%). The adjusted odds ratio was significant when age, sex, BMI, and smoking were adjusted (AOR = 2.48, 95% CI: 1.10–5.60, *p* < 0.05), whereas it turned insignificant when the working conditions were additionally adjusted (AOR = 2.28, 95% CI: 0.97–5.34, *p* < 0.1). The other psychosocial job characteristics did not show significant relationships with the onset of LBP after one year.

### Comparison of the baseline characteristics between those who did and did not participate in the follow-up survey

Among those with LBP at baseline, those who did not participate in the follow-up survey reported low social support at baseline more frequently than those who did (69.5% vs. 53.7%, *p* < 0.05). The other characteristics did not show statistically significant differences.

Among those without LBP at baseline, regarding the effect of LBP on work, those who did not participate in the follow-up survey reported “I do not have severe pain.” more frequently than those who did not (69.1% vs. 63.6%, *p* < 0.05). The other baseline characteristics, including all the psychosocial work characteristics, did not differ between those who did and did not participate in the follow-up survey.

## Discussion

We examined whether psychosocial work characteristics were associated with LBP in a sample of Japanese daycare workers. The prospective cohort analysis showed that low social support affected the persistence, but not the onset of LBP one year later. The other psychosocial work characteristics, i.e., high job strain, ERI, or overcommitment, did not show significant associations with LBP.

The findings of the longitudinal analysis suggest that low social support affected the persistence of LBP. It is generally essential for daycare workers to communicate well with their supervisors and coworkers while working, for example, when caring for the children and preparing meals, and for avoiding accidents and injuries to the children. Thus, having low social support would be stressful for daycare workers to execute their work effectively and safely, causing a stress response such as LBP. Previous studies pointed out the adverse effect of low social support [30] and lack of communication with supervisors and coworkers [31] on the psychological distress of daycare workers in Japan. Low social support could be more critical for daycare workers than for other kinds of workers. In contrast, the previous findings of prospective cohort studies [13–16], which included employees of other kinds of occupations as subjects, were not consistent regarding the effect of low social support on LBP. This may be because the level of social support required while working differs by occupation. In addition, we failed to find that low social support affected the onset of LBP among daycare workers in the present study. It is necessary to examine the impact of social support on LBP in more detail not only for daycare workers but also for other kinds of workers.

None of high job strain, ERI, or overcommitment were related to the persistence or onset of LBP. This could be because of the low prevalence of and the small numbers of subjects with high job strain, ERI, and overcommitment at baseline. Larger sample size could have been ideal for the present study. On the other hand, prospective cohort studies revealed that high job strain did not affect the persistence [13, 15–17] or onset of LBP [14] in other kinds of workers. As we already introduced, a prospective study by Koch et al. indicated that ERI was related to LBP in childcare workers in Germany [18]. This is the only study applying the ERI model to examining determinants of LBP in daycare workers. It must be noted that the prevalence of ERI at baseline was much higher in their study than that in our study: 65% vs. 8%. The psychosocial work environment was much different between their and our studies. Inconsistency was reported regarding the effect of ERI on the onset of LBP in other kinds of workers [19, 20]. No study has addressed whether overcommitment had a long-term effect on the persistence and onset of LBP among workers including daycare workers. More findings are necessary to clarify the effects of high job strain, ERI, and overcommitment on the persistence and onset of LBP among daycare workers.

We must discuss the pain intensity evaluated with the NRS score, the disability in daily life evaluated with the RDQ score, and the impact of LBP on work of the subjects. The NRS and RDQ scores of our subjects with LBP at baseline were lower than those of the patients with LBP [24, 32] and even the community dwellers with LBP [33]. Regarding the impact on work, approximately three-quarters of the subjects with LBP at baseline felt slight pain occasionally. That is, our subjects suffered from a low severity of LBP. Those whose LBP was too severe to work would not have participated in the present study. Therefore, the effects of psychosocial work characteristics on LBP could have been underestimated.

We here discuss our definition of LBP. First, we did not confirm the validity of the question “Where are you currently feeling LBP?” that we used to define LBP in our study. Researchers have long been discussing a methodological concern that the definition of LBP often differs by research [34]. Dionne et al. proposed a minimal definition that covered the site, symptoms, time frame, and severity of LBP [34]. The question and its answer options that we used in the present study covered the site, symptoms, and time frame. Since the respondents simply answered “yes” or “no” to each option, it was difficult to find the severity by this question. However, we evaluated the pain intensity, disability in daily life due to LBP, and impact of LBP on work of those with LBP, using other items. Therefore, we believe that our definition of LBP was valid and in line with the standard of relevant research. Second, there are two types of LBP, according to their duration: acute and chronic LBP [35]. Most LBP is acute, lasting a few days to a few weeks. Chronic LBP is defined to continue for 12 weeks or longer, even after an initial injury or underlying cause of acute LBP has been treated. In the present study, we did not ask the participants when their LBP occurred or how long the LBP lasted. We could not distinguish acute and chronic LBP in our studies. Persistent LBP might not mean chronic LBP. It was possible that those with LBP at baseline once recovered from LBP after a while but acute LBP recurred at follow-up.

There are some limitations to interpret our findings. First, our subjects only included those workers who were present on the day of the survey, and therefore not those absent due to sickness (or other reasons). It may have been the case that absent staff had taken leave due to LBP, representing a group of individuals with a more severe condition. Second, the non-participation rates of the follow-up study must be considered, i.e., 22.6% for the participants with LBP at baseline and 12.6% for those without. Among the participants with LBP at baseline, those who did not participate in the follow-up survey reported low social support more frequently than those who participated (69.5% vs. 53.7%, p < 0.030). This could underestimate the prospective effects of low social support on the persistence of LBP. Finally, we did not specify the cause of LBP in this study. Most cases would be attributable to the musculoskeletal system; however, there might be exceptions.

## Conclusion

We tested the association between psychological characteristics on LBP among daycare workers in Japan. Low social support was associated with the persistence of LBP, but not with the onset of LBP. None of high job strain, effort-reward imbalance, and overcommitment were associated with the persistence or onset of LBP.

**Table 3:** Relationships between psychosocial work characteristics in 2015 and persistent low back pain (LBP) in 2016 among participants with LBP in 2015 (*n* = 208)

Psychosocial work characteristics in 2015	LBP prevalence in 2016	Odds ratios and 95% confidence intervals for persistent LBP		
		Crude	Adjusted	
			Model 1	Model 2
**High job strain** ^**a**^				
** Absent**	**81.4% (118/145)**	**1**	**1**	**1**
** Present**	**91.5% (54/59)**	**2.47 (0.90–6.77)**	**2.65 (0.86–8.13)**	**2.48 (0.79–7.75)**
**Low social support** ^b^				
** Absent**	**80.0% (76/95)**	**1**	**1**	**1**
** Present**	**89.9% (98/109)**	2.23 (1.00–4.96)*	**2.13 (0.90–5.07)**	2.43 (1.01–5.87)*
**Effort-reward imbalance** ^c^				
** Absent**	**84.0% (147/175)**	**1**	**1**	**1**
** Present**	**88.2% (15/17)**	**1.43 (0.31–6.60)**	**1.27 (0.26–6.15)**	**1.40 (0.28–6.96)**
**Overcommitment** ^d^				
** Absent**	**82.5% (113/137)**	**1**	**1**	**1**
** Present**	**87.7% (57/65)**	**1.51 (0.64–3.58)**	**1.46 (0.58–3.68)**	**1.51 (0.58–3.93)**

Model 1: adjusted for age, sex, BMI, and smoking in 2015

Model 2: adjusted for age, sex, BMI, smoking, regular employment, occupation, and irregular work in 2015

The numbers of missing responses: a) 4, b) 4, c) 16, and d) 6

*p<0.05

**Table 4:** Relationships between psychosocial work characteristics in 2015 and onset of low back pain (LBP) in 2016 among participants without LBP in 2015 (*n* = 150)

Psychosocial work characteristics in 2015	LBP prevalence in 2016	Odds ratios and 95% confidence intervals for the onset of LBP		
		Crude	Adjusted	
			Model 1	Model 2
**High Job strain** ^a^				
** Absent**	**28.1% (34/121)**	**1**	**1**	**1**
** Present**	**37.0% (10/27)**	**1.51 (0.63–3.61)**	**1.95 (0.77–4.95)**	**2.36 (0.88–6.30)**
**Low Social support** ^b^				
** Absent**	**24.4% (22/90)**	**1**	**1**	**1**
** Present**	**37.9% (22/58)**	**1.89 (0.92–3.86)**	2.48 (1.10–5.60)*	**2.28 (0.97–5.34)**
**Effort-reward imbalance** ^c^				
** Absent**	**31.1% (41/132)**	**1**	**1**	**1**
** Present**	**18.2% (2/11)**	**0.49 (0.10–2.39)**	**0.69 (0.13–3.63)**	**0.46 (0.07–3.01)**
**Overcommitment** ^d^				
** Absent**	**27.1% (29/107)**	**1**	**1**	**1**
** Present**	**39.5% (15/38)**	**1.75 (0.81–3.82)**	**1.63 (0.70–3.76)**	**1.45 (0.60–3.50)**

Model 1: adjusted for age, sex, BMI, and smoking in 2015

Model 2: adjusted for age, sex, BMI, smoking, regular employment, occupation, and irregular work in 2015

The numbers of missing responses: a) 2, b) 2, c) 7 and d) 5

*p<0.05

## Data Availability

Upon a reasonable request, the corresponding author will share the data used in this study after approval of the Institutional Review Board of Fujita Health University, following the Ethical Guidelines for Medical and Biological Research Involving Human Subjects established by the Ministry of Education, Culture, Sports, Science, and Technology, the Ministry of Health, Labor, and Welfare, and the Ministry of Economy, Trade, and Industry, Japan.

## Abbreviations

AOR: Adjusted odds ratio
BMI: Body mass index
ERI: Effort-Reward Imbalance
JDCS: Job Demand Control Support
LBP: Low back pain
NRS: Numerical rating scale
RDQ: Roland-Morris Disability Questionnaire
